# An Atypical Presentation of Progressive Multiple Sclerosis in a Young Black Male

**DOI:** 10.7759/cureus.45496

**Published:** 2023-09-18

**Authors:** Charles Franks, Adi Eylon, Arturo Carrion, Ramon Bassa

**Affiliations:** 1 Dr. Kiran C. Patel College of Osteopathic Medicine, Nova Southeastern University, Fort Lauderdale, USA; 2 Internal Medicine, Reception and Medical Center, Lake Butler, USA

**Keywords:** demyelinating disorders, primary progressive multiple sclerosis, ms, neurology, phasic spasticity, progressive multiple sclreosis, correctional medicine, multiple sclerosis

## Abstract

Multiple sclerosis (MS) is an autoimmune disease primarily affecting the central nervous system, commonly diagnosed in women and individuals of European ancestry. It most commonly presents in the form of relapsing-remitting MS, which is characterized by exacerbations with partial to complete recovery. Far less common is the primary progressive form of MS, which involves the progression of neurological symptoms that gradually worsen with time. We present an atypical case of progressive MS in a 26-year-old incarcerated Black male. Initially diagnosed in 2019, he experienced bilateral upper extremity weakness and phasic spasticity, with subsequent worsening of symptoms including lower extremity spasticity, vision impairment, and difficulties with mobility and writing. With progressing symptoms, unintentional weight loss, and declining motor function, he was admitted to the hospital in March 2023. This case emphasizes the importance of considering MS as a differential diagnosis in any patient with progressive neurological dysfunction because, unlike the more prevalent relapsing-remitting type of MS, primary progressive MS has a more insidious onset with no recovery between exacerbations. It addresses the patient's symptom history, medication compliance challenges, and the need for improved education and awareness of MS in diverse patient populations.

## Introduction

Multiple sclerosis (MS) is the most common immune-mediated inflammatory demyelinating disease of the central nervous system, ultimately leading to the destruction of the myelin sheath that surrounds and protects nerve fibers. MS is a clinical diagnosis, and MRI is the test of choice to support the clinical diagnosis of MS [1]. The core phenotypes of MS are relapsing-remitting and progressive disease. Relapsing-remitting MS is the most common phenotype at disease onset, even more so in young people. It is characterized by exacerbations and relapses with full or partial recovery and accounts for 85-90% of MS cases [2]. Relapsing-remitting MS can become secondary progressive MS, which is defined by a steady progression of the disease course and loss of periods of remission. Primary progressive MS, which only accounts for approximately 10% of MS cases, is characterized by progressive accumulation of disability from onset with only minor improvements, if any. Primary progressive MS is diagnosed through patient history, as no imaging or exam findings will distinguish primary from relapsing-remitting disease [3]. To satisfy the McDonald criteria, a patient must have one year of clinical symptoms as well as two of three objective findings including at least one hyperintense T2 lesions characteristic of MS in the periventricular, cortical, juxtacortical, or infratentorial areas; at least two hyperintense T2 lesions in the spinal cord; and presence of oligoclonal bands in the CSF [4]. In primary progressive MS, the most common clinical presentation is a spinal cord syndrome that worsens over months or years with asymmetric spastic paraparesis and no clear sensory level [5]. Less often, primary progressive MS develops as a progressive cerebellar ataxia and rarely with cognitive, brainstem, or visual symptoms.

There are no clinical manifestations that are unique to MS, but some are highly characteristic of the disease. Common symptoms and signs of MS include sensory symptoms in the limbs or one side of the face, visual loss, acute or subacute motor weakness, diplopia, gait disturbance and balance problems, Lhermitte sign (electric shock-like sensations that run down the back and/or limbs upon flexion of the neck), vertigo, bladder problems, limb ataxia, acute transverse myelitis, and pain [6]. Spasticity is a common symptom of MS and is characterized by increased muscle tone due to an upper motor neuron lesion caused by demyelination of the corticospinal system. Tonic spasticity is characterized by resistance to movement that is rate-dependent, while phasic spasticity manifests as involuntary jerks and spasms that are more pronounced at night when attempting to sleep [7,8]. Tonic spasticity is generally more common in patients with MS than phasic spasticity [9]. Moreover, approximately 50% of patients with MS report bowel dysfunction, and up to 75% report bladder dysfunction [10]. Cross-sectional studies have shown some degree of affective disturbance in up to two-thirds of patients with MS [11]. Fatigue was the most common currently experienced symptom (86%), and it was rated as the worst symptom causing difficulty or distress by 65%, higher than any other symptom [6]. MS classically affects females more often than males. A systematic review of epidemiologic studies from 1955-2000 revealed an estimated female-to-male ratio between 1.4:1 and 2.3:1 [12]. MS has also been classically known to disproportionately affect White individuals of European background; however, recent literature demonstrates similar incidence rates among White and Black individuals, primarily females.

Here, we report a case of MS in a young Black male, which is unusual due to the patient's gender, age at onset, and symptomatology. Of note, the extent of the patient's disease and responsiveness is not fully understood as our patient did not report all of his symptoms throughout his disease course and was not fully compliant with treatment regimens. Our case presentation will consist of some information reported by our patient, but the majority of information is gathered from available clinical notes and lab reports.

## Case presentation

Our patient is a 26-year-old incarcerated Black male, first diagnosed with MS in 2019. At the age of 23, he began experiencing bilateral upper extremity weakness and spastic hypertonia, worse on the right side. No imaging is available from this time, but a lumbar puncture from December 2019 showed a Myelin basic protein of 6.16 mg/ml (normal range: 0.00-5.50 mg/ml). Throughout 2020-2021, our patient began to experience lower extremity weakness and spastic hypertonia, as well as worsening vision in his right eye which he described as “dim and blurry.” In October 2021, our patient saw an optometrist who noted a left eye sphere of -0.25, a cylinder of -1.50, and an axis of 175. No nystagmus was noted during this evaluation, and extraocular movements were intact. The consulting optometrist found that the patient had no remaining functional use of his right eye, with a visual acuity of less than 20/200.

In December 2021, our patient reported to an urgent care setting within the Department of Corrections, reporting worsening cerebellar ataxia and spastic hypertonia, making walking and writing difficult. He believed these symptoms to be related to two retained bullets from a gunshot wound he suffered before incarceration. It should be noted that while our patient does have superficial evidence of gunshots near the left nipple and right axilla, he has since received multiple X-rays, none revealing any retained foreign objects.

In February 2022, our patient started on the immunomodulator glatiramer acetate (Glatopa) at a dose of 40 mg/mL three times per week. Later this month, he reported that the medication led to worsening shaking and difficulty with fine motor coordination. At this time, he still believed he was misdiagnosed with MS and refused all future Glatopa doses, relating his neurological symptoms with retained bullets. By March 2022, our patient noted trouble eating as he could not chew properly, and he now required a wheelchair to ambulate due to profound lower extremity weakness and spastic hypertonia.

In May 2022, our patient requested that a physician put him on a “nerve medication” to help with his symptoms, as he was losing weight due to an inability to eat or swallow properly. Between December 2021 and May 2022, our patient’s weight decreased from 182 lbs to 169 lbs, an unintentional weight loss of 13 pounds. In May 2022, our patient saw his physician, who noted 5/5 strength in the left lower extremity, 4/5 strength in the left upper extremity, 5/5 strength in the right lower extremity, and 3/5 strength in the right upper extremity. He also noted horizontal nystagmus, positive Romberg sign, dysmetria in the bilateral upper and lower extremities, and spastic gait. The physician's recommendations were to attain an MRI of the brain with and without contrast, an MRI of the cervical spine with and without contrast, as well as to restart Glatopa.

Our patient continued to refuse disease-modifying agents but followed up with neurology in June 2022, complaining of shortness of breath when talking and feeling depressed. In July 2022, our patient was placed in isolation due to a positive rapid COVID-19 test, but he did not complain of any COVID-19-related symptoms at this time. In August 2022, he asked to start Glatopa due to worsening weakness, incoordination, and spastic hypertonia but continued to sign refusals every time he was offered the medication. In September 2022, he complained of frequent urination which he attributed to his bladder feeling “overactive.” Throughout the rest of 2022, our patient continued to present to the urgent care setting complaining of diffuse weakness, spastic hypertonia, and difficulty with activities of daily living. Even with continued education on his diagnosis during every visit, he refused treatment with a disease-modifying agent.

In February 2023, our patient underwent a brain MRI, complicated by spastic hypertonia which led to continued movement throughout the imaging. The radiologist identified severe demyelination in the periventricular and deep white matter, within the brainstem (including the midbrain, pons, and medulla), and within the basal ganglia. Some demyelination was also noted in the bilateral cerebellar hemispheres. No focal-enhancing lesions were appreciated during this imaging.

In March 2023, our patient followed up with a neurologist for dysarthria, difficulty ambulating, frank tremors, and decreased motor effort in bilateral extremities. It was recommended that our patient be evaluated by a MS specialty clinic and begin physical therapy. It was also recommended to start Gabapentin and a muscle relaxant for symptomatic relief of his spastic hypertonia and associated pain.

March 2023 is when the patient presented to our inpatient facility. On physical exam, it was immediately evident that this patient had no retained fine motor skills and displayed movement consistent with severe phasic spasticity. Even gross motor movement was poorly controlled, resulting in the patient requiring assistance with activities of daily living including feeding, bathing, transferring, and toileting. Cranial nerves I and III-XII appeared grossly intact. The patient's right eye had visual acuity worse than 20/200 utilizing the Snellen chart. Further workup is required to confirm whether cranial nerve II is the cause of this visual deficit. Left eye vision was intact with a visual acuity of 20/20. The patient did not display nystagmus during our oculomotor testing. A fundoscopic exam was deferred during this physical exam. The patient had a fully intact sensation of dull and sharp touch throughout his body. The patient was noted to have dysarthria and slow speech, but speech content was normal.

Our patient displayed profound dysdiadochokinesia, as well as bilateral dysmetria on an exam. Multiple practitioners were unable to elicit deep tendon reflexes due to the patient's severe dystonia and inability to relax his extremities. The plantar reflex was symmetrically mute. Grip strength was 5/5 bilaterally, but the patient became dystonic while gripping, leading to uncontrollable flailing movements followed by full arm extension until the grip was released. Bilateral pronator drift was also appreciated. The patient's lower extremities displayed 4/5 strength and also demonstrated dystonic movements when he attempted any movement. When interviewed, the patient described his symptoms as progressively worsening since his presentation to the facility and endorsed no periods of remission since onset. As of the time of writing, our patient is awaiting an MRI with and without contrast of the cervical spine and has a consult written for physical therapy. He is seen by providers daily, and while it is not evident yet if he will agree to disease-modifying therapy (DMT), he is hopeful of what physical therapy may offer.

## Discussion

This case is notable for several reasons not only pertaining to the patient’s demographics but also the clinical findings and progressive nature of its course. As stated in the introduction, relapsing-remitting is by far the most typical disease course for MS and accounts for 85-90% of MS cases [2]. Our patient displays a clinical history of primary progressive MS, which only accounts for approximately 10% of MS cases. The typical presentation of primary progressive MS (PPMS) consists of gradual progression, with steady worsening over time. A typical patient may have difficulty walking, spasticity (commonly in the form of tonic spasticity, marked by stiffness), and fatigue. Moreover, typical brain involvement with PPMS is localized in the juxtacortical and cortical areas [13]. A less typical presentation of PPMS involves a more sudden symptom onset that may mimic the attacks seen in RRMS, as well as phasic spasticity marked by clonus, cramps, and spasms. This atypical presentation may also include cognitive symptoms, such as difficulty concentrating and memory impairment, and atypical neurological symptoms affecting vision, speech, or other functions not commonly associated with MS. These unusual presentations may lead to misdiagnosis.

Our patient has a combination of typical and atypical symptom presentation, which made it difficult to diagnose initially. He has typical difficulty walking and fatigue. However, the onset of his symptoms was more rapid than normal, and he displayed profound phasic spasticity, which has been established as the less common form of spasticity in MS [9]. His initial symptoms were upper extremity weakness, spastic hypertonia, and decreased vision in his right eye. Within two years, he lost all visual functions of his right eye. It should be noted that although his myelin basic protein came back slightly elevated on initial evaluation, it took three years since his symptom onset for him to be diagnosed with PPMS and offered treatment. As a result of his disease progression, our patient's speech was disrupted and he suffers from dysphagia. Additionally, our patient was found to have extensive brainstem and cerebellar involvement, all features less typical of primary progressive MS [5]. These findings also point to a more aggressive disease, given that they implicate the brainstem.

The challenges posed by such an atypical presentation in establishing the diagnosis of MS are worth discussing, and they include the stereotypical versus actual demographic and clinical characteristics associated with the disease. In regard to age, although the mean age at diagnosis of relapsing-remitting MS is approximately 30 years, primary progressive disease is typically diagnosed at a mean age of 40, making our patient's age of 23 at disease onset atypical [2] and a possible contributor to his diagnosis delay. Moreover, while MS has been classically known to disproportionately affect white individuals of European background, recent incidence reports suggest an increasing rate of MS among African Americans. When correcting for sex and race, Black males tend to have a similar risk of MS compared to their White male counterparts, although this chance remains far less than for females of either race [14]. Furthermore, as previously stated, the female-to-male ratio of MS has increased since 1950 from 1.4:1 to 2.3:1 [12]. Recent studies have found that the incidence of MS in females is increasing, leading to a further increase in the female-to-male ratio [15]. It should be noted, however, that primary progressive MS, which is what our patient displays, has been shown to affect males and females equally, and oftentimes affecting males more than females [16]. Therefore, his gender alone does not make this case a rarity. However, in combination with his young age, Black race, and symptoms, his gender may have contributed to the diagnostic uncertainty early on. The patient's demographics and aggressive disease presentation (i.e., extensive cerebellum and brainstem involvement, phasic spasticity, vision impairment, dysphagia, severe limb weakness) may not seem like a diagnosis of MS to a healthcare provider that is not up to date on recent demographic findings pertaining to MS. MS has traditionally been thought to affect white individuals predominantly, and it was only recently proven that Black individuals are affected in similar rates. Therefore, it is important that healthcare providers encountering a patient similar to ours consider the high likelihood of MS and are aware of recent demographic data disproving the stereotype of an MS patient.

The treatment challenges and considerations for DMT in such atypical MS cases are important to mention because there are limited options that have been shown to be effective in patients like ours with advanced PPMS. The optimal treatment for PPMS is with ocrelizumab, the first FDA-approved drug for PPMS. It was shown to reduce disability progression and deterioration from baseline and improve MRI lesion findings and brain volume loss [17]. However, the primary study used to assess efficacy comprised primarily of younger patients and more active, early-stage PPMS, who may have an optimal response to the anti-inflammatory ocrelizumab effects. Therefore, its efficacy is unknown in a patient like ours who is far beyond the early stage. It is initially dosed as a 300 mg IV infusion and a second infusion two weeks later. After that, it is administered as a 600 mg IV infusion every six months. The infrequent dosing of this medication would improve patient compliance, especially in our patients. However, this medication has limited availability and high pricing, which may likely deter a patient like ours from seeking treatment.

There are other DMTs used in RRMS that have not shown evidence of benefit in PPMS, such as fingolimod, glatiramer acetate (which is what our patient was given), interferons, and rituximab. Our patient's poor response to therapy can be due to the lack of beneficial evidence in its use for PPMS, in addition to his poor medication compliance. Other alternative medications that are used empirically in PPMS with a lack of sufficient evidence demonstrating their effectiveness include glucocorticoids, methotrexate, IV immune globulin, and mitoxantrone. It is important to be aware of these options, though they are limited, lack promising evidence, and are, therefore, not often utilized for PPMS. Discussing these treatment strategies for atypical presentations is valuable because it can assist in the optimization of management for similar complex cases. Although the progression of our patient's disease may have been slowed if he agreed to remain on immunomodulating therapy, his response to therapy cannot be properly discussed due to poor medication compliance. Had he been more compliant, it would have been easier to determine whether the glatiramer acetate was helping in order to optimize his treatment with the best medication for him. In addition, while current research has shown that early initiation of DMT is associated with a decrease in the short-term progression of MS [18], further research is needed to evaluate the efficacy of DMT in decreasing the long-term progression of MS [19].

Finally, regarding the patient's concerns regarding his gunshot wounds causing MS, current literature shows that physical trauma is not related to disease induction or relapse [20].

## Conclusions

This case highlights the importance of considering MS in the differential diagnosis of young Black males with atypical neurological symptoms. Even if a patient does not fit the classic clinical vignette classically associated with a disease process, early identification and treatment can lead to a more favorable prognosis. As discussed, classically accepted trends of MS demographics are being challenged by current research. There is a need for continued research into the features of MS in individuals of African descent, including unique clinical presentations and responses to therapy.
